# Metagenomic analysis of the rumen microbial community following inhibition of methane formation by a halogenated methane analog

**DOI:** 10.3389/fmicb.2015.01087

**Published:** 2015-10-13

**Authors:** Stuart E. Denman, Gonzalo Martinez Fernandez, Takumi Shinkai, Makoto Mitsumori, Christopher S. McSweeney

**Affiliations:** ^1^CSIRO, Agriculture Flagship, Queensland Bioscience PrecinctSt. Lucia, QLD, Australia; ^2^National Institute of Livestock and Grassland ScienceTsukuba, Japan

**Keywords:** rumen, methane, H_2_, microbial community, metagenomics

## Abstract

Japanese goats fed a diet of 50% Timothy grass and 50% concentrate with increasing levels of the anti-methanogenic compound, bromochloromethane (BCM) were investigated with respect to the microbial population and functional shifts in the rumen. Microbial ecology methods identified species that exhibited positive and negative responses to the increasing levels of BCM. The methane-inhibited rumen appeared to adapt to the higher H_2_ levels by shifting fermentation to propionate which was mediated by an increase in the population of H_2_-consuming *Prevotella* and *Selenomonas* spp. Metagenomic analysis of propionate production pathways was dominated by genomic content from these species. Reductive acetogenic marker gene libraries and metagenomics analysis indicate that reductive acetogenic species do not play a major role in the BCM treated rumen.

## Introduction

Enteric fermentation in ruminants generates methane, which can represent an energy loss of between 2–12% for the animal (Johnson and Johnson, 1995) and significantly contributes to greenhouse gas emissions (Hristov et al., 2013). Significant efforts are being made to develop nutritional strategies, based on the use of antimethanogenic compounds, to reduce methane production in ruminants (Benchaar and Greathead, 2011). In this metabolic process, H_2_ obtained from the conversion of feedstuffs into various fermentation end products is mainly consumed by methanogens to produce methane in the rumen (Janssen, 2010). Therefore, H_2_ produced in the rumen has to be considered when developing strategies to control ruminant methane emissions since H_2_ accumulation can impair digestion and fermentation if it accumulates (Wolin et al., 1997). Furthermore, redirecting rumen H_2_ production toward alternative energy yielding pathways could improve efficiency of energy utilization from feed in ruminants.

Ungerfeld and Kohn (2006) provided an excellent overview of the thermodynamics of ruminal fermentation and identified several strategies for utilizing H_2_ in the rumen as an alternative to methanogenesis. Fumarate and malate have been used by several investigators to stimulate succinate/propionate producers in the rumen which compete with methanogens for H_2_. Many of these organisms use the succinate–propionate (randomizing) pathway as a major route for propionate synthesis in the rumen (Baldwin et al., 1963). In this pathway, malate is dehydrated to fumarate, and the reduction of fumarate to succinate is coupled to ATP synthesis. Succinate is either an intermediate or an end-product in the pathway of different rumen bacteria. However, several researchers have shown in mixed ruminal cultures that fumarate (and malate) is converted to propionate and acetate in varying proportions (Ungerfeld et al., 2007). The relative amounts of propionate and acetate formed from fumarate will impact on the H_2_ pool available to methanogens. Stoichiometrically, propionate production from fumarate consumes one pair of reducing equivalents while acetate production from fumarate releases two pairs of reducing equivalents. Therefore, the production of acetate from fumarate is counterproductive when the objective is to reduce H_2_ available for methane production. It is important therefore to identify the microorganisms involved in these pathways and determine the physiological and biochemical conditions which favor propionate rather than acetate production from fumarate.

Bromochloromethane-cyclodextrin (BCM-CD) has been used in numerous studies to reduce methane production in ruminants and it is considered one of the more effective methane inhibitors (Denman et al., 2007; Goel et al., 2009; Tomkins et al., 2009; Abecia et al., 2012; Mitsumori et al., 2012). The responses to the BCM-CD of rumen fermentation, methane production, H_2_ flux and microbial abundances have been previously published (Mitsumori et al., 2012), showing a dose-dependent inhibitory effect on methane production and an increase in H_2_ production, with no detrimental effects on rumen fermentation. In line with other studies, there was an observed fermentation shift toward more propionic production, which could be due to a reduction in competition for H_2_ in the rumen. Real-time PCR quantification of microbial groups showed a decrease in abundance of methanogens and fungi, whereas there were an increase in *Prevotella* spp. and *Fibrobacter succinogenes* (Mitsumori et al., 2012). It was concluded that the methane-inhibited rumen adapted to the higher H_2_ levels by shifting fermentation to propionate via *Prevotella* spp., but the majority of metabolic H_2_ was expelled as H_2_ gas (Mitsumori et al., 2012). However, the effect on microbial populations has not been studied in depth, particularly in those microorganisms that play an important role in hydrogenotrophy pathways when methanogenesis has been impeded in the rumen. New molecular techniques, such as next-generation sequencing (NGS), have been adopted to study rumen microbiology, providing a higher resolution observation of the rumen microbial populations with respect to metabolic activity, abundance and facilitating the analysis of an increased volume of data (Callaway et al., 2010; Hess et al., 2011; Lee et al., 2012; Pope et al., 2012; Ross et al., 2012; St-Pierre and Wright, 2013).

Therefore, we have studied the effect of different levels of the antimethanogenic compound, BCM-CD, on the rumen microbial community in goats using metagenomic sequence analysis. The aim of the present study was to identify and characterize the microbiology and genetics underpinning the alternative hydrogenotrophic pathways in ruminants, using metagenomics.

## Materials and Methods

### Animals and Experimental Design

Three ruminally fistulated Japanese native goats (*Capra aegagrus hircus*, female) 35.7 ± 4.85 kg were treated with three levels of the antimethanogenic compound BCM.

The experimental design, treatments and fermentation parameters have been described by Mitsumori et al. (2012). Animals were initially adapted to the basal diet for 14 days and measured as a control period. Within the sampling period, goats were placed into the respiration chambers for a period of 3 days for analysis of rumen gas production. Animals were then adapted to the increasing doses of BCM for 8 days before sampling in the respiration chambers for a further 3 days. Rumen samples were collected at the end of each respiration-sampling period (3 days) for DNA extractions. The animal experiments were carried out in accordance with a protocol approved by the Guide for the Care and Use of Experimental Animals (Animal Care Committee, NILGS).

### DNA Extractions

DNA extractions were performed on rumen samples collected from the goats using the FastDNA kit and FastPrep instrument (MP Biomedicals, Cleveland, OH, USA; Mitsumori et al., 2012).

### Functional Gene Analysis

Functional gene libraries were constructed from DNA samples collected from the control and high BCM dosing periods. One, targeting methanogenesis using the methyl coenzyme A reductase (mcrA), using mcrA forward primer 5′-GGTGGTGTMGGATTCACACARTAYGCWACAGC and mcrA reverse primer 5′-TTCATTGCRTAGTTWGGRTAGTT (Luton et al., 2002). Two libraries targeting reductive acetogenesis genes: the formyl tetrahydrofolate synthase (FTHFS), using the FTHFS forward primer 5′-TTYACWGGHGAYTTCCATGC-3′; and FTFHS reverse 5′-GTATTGDGTYTTRGCCATACA-3′ (Leaphart and Lovell, 2001) and acetyl CoA synthetase (ACS), using the ACS forward primer 5′-CTBTGYGGDGCIGTIWSMTGG and ACS reverse 5′-AARCAWCCRCADGADGTCATIGG (Gagen et al., 2010). Amplified PCR products were gel extracted from a 1.5% agarose gel and cloned using the pGEM-Teasy vector system (Promega). Transformed *Escherichia coli* cells harboring the cloned products were selected and sequenced using BigDye sequencing reagents (ABI). Sequence data was analyzed and placed in phylogenetic trees in the ARB software environment (Ludwig et al., 2004) and clustering to a defined operational taxonomic unit (OTU) analysis performed using MOTHUR (Schloss et al., 2009).

### 16S rDNA Analysis

16S rRNA gene pyrotagging was performed using modified universal bacterial primers (27f and 515r; Lane, 1991; Felske et al., 1997). Specific sequences matching the Roche 454 sequencing adaptor B were added to the 27f primer, while adaptor A was added to the 515r. In addition between the adaptor A sequence and the 16S 515r sequence a 10 bp barcode was inserted. Each individual DNA sample was amplified using the 27f primer and a uniquely barcoded 515r primer. After, amplification products were visualized by performing gel electrophoresis. Product quantities were calculated and an equal molar amount of each product was pooled. The pooled products were run in a 1.5% agarose gel and the product gel extracted and purified prior to submission for 454 pyrosequencing.

Short read sequence data generated using 454 sequencing was analyzed using the QIIME: Quantitative Insights Into Microbial Ecology software package (Caporaso et al., 2010). Raw sequences were passed through Acacia for 454 error correcting (Bragg et al., 2012). Error corrected sequences were then de-multiplexed in QIIME based on their unique barcode, clustering of sequences to OTUs of 97% similarity were performed using uclust (Edgar, 2010). Chimeric sequences were identified using chimera slayer (Haas et al., 2011) and removed. Taxonomic assignment of sequences was performed against the Greengenes database (McDonald et al., 2012) using the RDP classifier software (Wang et al., 2007).

Additional analysis of OTU’s was performed in the R packages ade4 and Phyloseq (Chessel et al., 2004; McMurdie and Holmes, 2013). The sequences obtained in this paper have been deposited in the European Nucleotide Archive (ENA) under the accession number PRJEB10560.

### Meta-genomic Analysis

Metagenomic assessment of the goat microbiome from the control and high BCM dosing using 454 pyro-sequencing was undertaken. DNA extracted from the three goats for control and high BCM samples were pooled based on treatment, control and BCM respectively and nebulized and 454 adapter fragments were added. One half plate of sequencing was performed on each library, data generated from the 454 sequencing run was initially passed through CD-HIT for de-replication of the 454 data (Niu et al., 2010). De-replicated reads were analyzed for the occurrence of ribosomal DNA reads using hidden Markov models implemented in the software package hmm_rRNA (Huang et al., 2009). Phylogenetic analysis of metagenomics samples for the comparison of community structures was performed using PhyloSift (Darling et al., 2014). Normalization of read data for functional abundance profiling was performed by calculating reads per kb per genome equivalent after accounting for the estimated average genome size of the microbial communities in each sample using MicrobeCensus (Nayfach and Pollard, 2014). Assembly of metagenomic sequences into larger contiguous sequences (contigs) was implemented with Newbler (Roche ver. 2.6). Contigs and orphaned reads were annotated using the MG-RAST server (Meyer et al., 2008), phylogenetic placement of reads and contigs was performed using both PhyloPythiaS (Patil et al., 2012) and RAIphy (Nalbantoglu et al., 2011). Sequence data has been deposited at MGRAST under project number 14718 (MG-RAST ID for BCM sample 4452826.3 and Control 4452824.3).

## Results

### 16S rDNA “Pyrotag” Monitoring of Microbial Populations

As previously described, a dose mediated response was observed for goats that were administered increasing concentrations of the anti-methanogenic compound BCM (Mitsumori et al., 2012). With increasing concentrations of BCM, a decrease in the measured methane output was detected with an increase in H_2_ release and fermentation shifts toward propionate production. Preliminary analysis of the rumen microbial populations using denaturing gradient gel electrophoresis (DGGE) and quantitative PCR (qPCR) found changes in fibrolytic species and an increases in certain *Prevotella* OTUs (Mitsumori et al., 2012).

**FIGURE 1:**
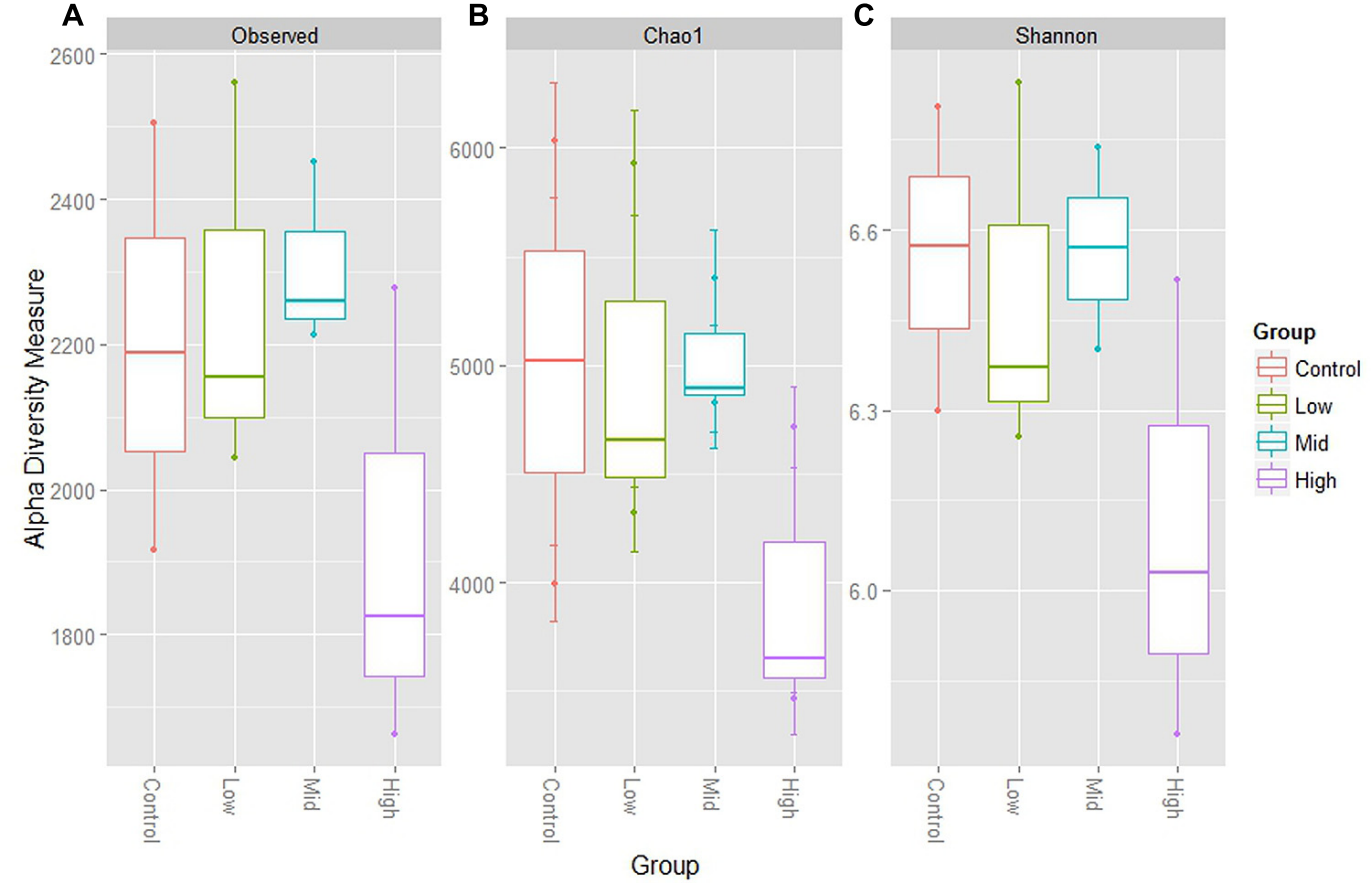
**Alpha diversity measures for goat rumen microbiomes at control, low, mid, and high doses of BCM. (A)** Shows total observed taxonomic units, **(B)** the Chao1 estimates and, **(C)** the Shannon diversity index. Boxplots indicate the first and third quartiles with the median value indicated as a horizontal line the whickers extend to 1.5 times the inter quartile range.

**FIGURE 2:**
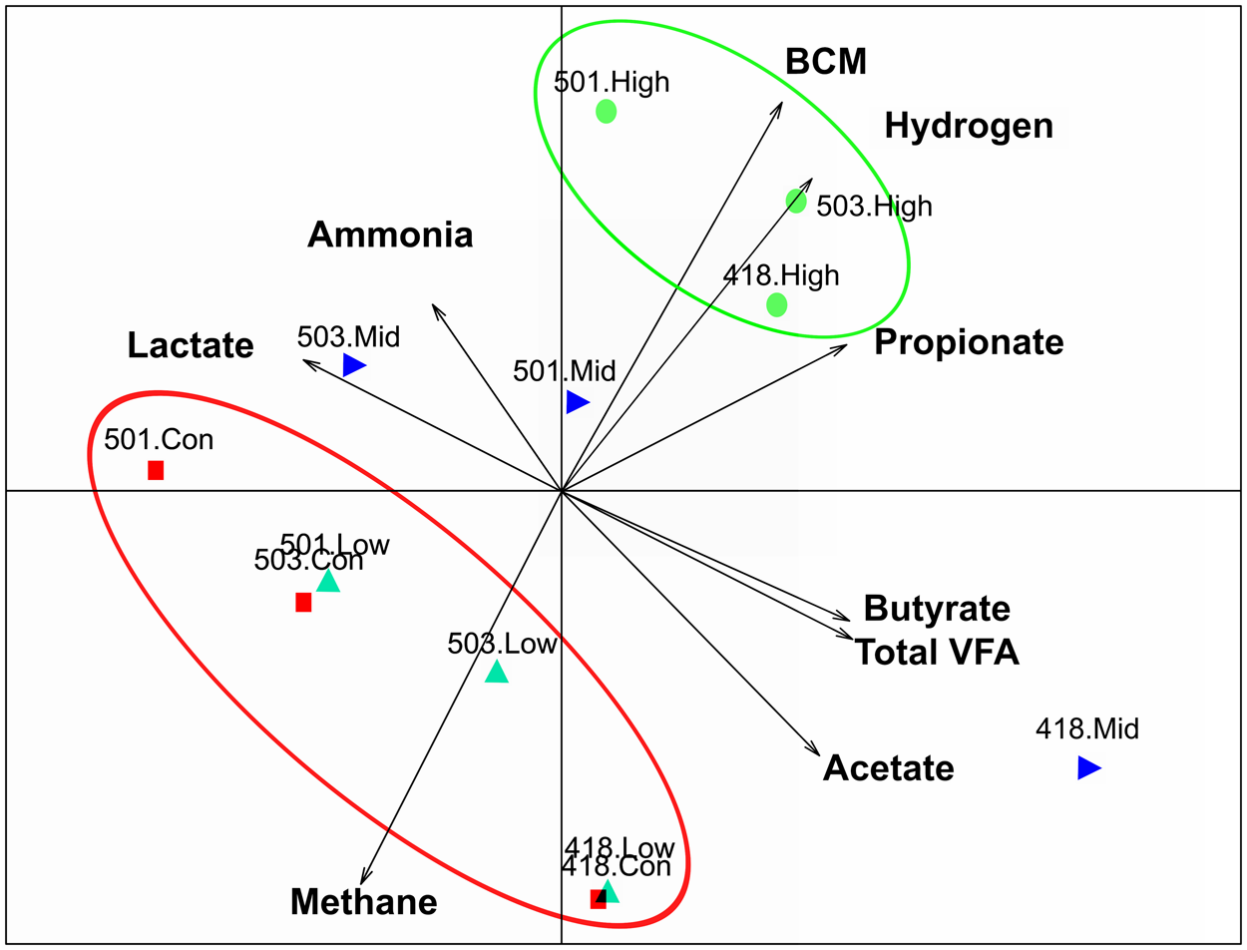
**Principal coordinate analysis co-inertia plot coupling rumen microbiome Euclidean analysis and biochemical measures, with arrows indicating increasing values of biochemical measures.** Rumen microbiomes for animals on varying levels of BCM for control (red square), low dose (turquoise triangle), mid dose (blue right triangle), high dose (green circle), labels indicate animal number and treatment.

An extensive analysis of the rumen microbiota showed that total microbial species richness of the goat rumen microbiome was not impacted with the administration of the low and mid doses of BCM. With no significant changes to alpha diversity [Shannon 6.56 ± 0.15, 6.49 ± 0.18, 6.57 ± 0.10 for Control, low and mid dose, respectively (mean ± SEM)]. While the highest dose of BCM caused a contraction in observed and estimated species richness (Shannon 6.10 ± 0.22) with an ∼14–20% decrease, respectively (**Figure 1**). The structure of the microbiomes as assessed by beta diversity measures (unifrac) clearly showed alterations between the control, low and mid, high dose groups, with 28% of the variance being explained between the control and highest dose of BCM (**Figure 2**). Variation in the rumen microbiome between animals explained the next level of variance with one animal possessing a divergent microbial population compared to the other two animals (**Figure 2**). The variance between the animals was less at the highest dose of BCM compared to all other treatments. Co-inertia analysis applied to the microbiome and biochemical measures of the rumen samples placed the high BCM microbiomes on the axis coupled with increasing BCM, propionate and H_2_ with a concurrent decrease in methane, while the control and low BCM dose were on the opposite axis (**Figure 2**). The goat rumen microbiome for the control diet was dominated by OTUs assigned to the Bacteroidetes and Firmicutes phylum (60 and 24%, respectively; Supplementary Figure S1). Both the Synergistetes and Lentisphaerae phylum contributed ∼4% each to the microbiome of control animals. An increase in the proportion of sequences assigned to the Bacteroidetes phylum was observed for the increasing doses of BCM with a concomitant decrease in Firmicutes, Synergistetes, and Lentisphaerae. Seventy eight OTUs were positively correlated (*r* > 0.6, *p* < 0.05) to the increased concentration of BCM administered to the animal, accounting for 27.88% of the sequence data at the high BCM samples with 52 of these assigned to the *Prevotella* genus level (Supplementary Figure S2). The OTUs that corresponded to the previously described *Prevotella* groups 1 and 7 from DGGE analysis (Mitsumori et al., 2012) were identified and were positively correlated with the increase in BCM concentration *r* = 0.997 and 0.999 respectively. The observed fold change for the sequence data was generally consistent with that of the qPCR data as previously reported for these two *Prevotella* groups (Mitsumori et al., 2012). Sequence abundance data found that these species were observed on average at 6.5 and 9.4% of the species sequenced respectively for the high BCM samples. Nine OTUs of lower abundance from the family Veillonellaceae were positively associated with increasing BCM concentrations (*r* = 0.61–0.79), of which six were further characterized to belong to the genus *Selenomonas.* These OTUs accounted for 0.78% of the sequence data in the high BCM samples. A single OTU accounting for 0.9% of the sequence data in the high BCM samples was associated with the genus *Succiniclasticum* and was moderately associated with the change in BCM levels (*r* = 0.52). Sequence data classified to OTUs closely associated to the fiber degrading species *F. succinogenes*, although observed to significantly increase at the mid and high dose of BCM, were only moderately correlated with a dose response of BCM (*r* = 0.30–0.59).

The increasing level of BCM in the rumen was negatively associated (*r* < -0.6, *p* < 0.05) with 27 OTUs of which six were assigned to the TG5 genus of the phylum Synergistetes (1.49% abundance of control sample), all of which were not observed at the highest BCM level (Supplementary Figure S2). A further five from the BS11 family from the Bacteroidetes phylum and three associated with Victivallaceae family from the Lentisphaerae phylum were also not detected at the high BCM levels.

### Hydrogenotrophic Functional Gene Analysis

Gene libraries encoding functional genes associated with methanogenesis (methyl coenzyme-M reductase, mcrA, Supplementary Figure S3) and reductive acetogenesis (FTHFS, Supplementary Figure S4; ACS, Supplementary Figure S5) were constructed from control and high BCM samples.

Diversity of methanogens from the control goat rumen were dominated by members of the Methanobacteriales family. The administration of BCM produced a marked decrease in the methanogen diversity compared to the control period with only 27% of the sequence data shared between the two samples. The predominant OTU in the control and high BCM were different, however the dominant species in both were found to be affiliated with *Methanobrevibacter* species.

Investigations related to the diversity of the FTHFS amino acid sequences from the *fhs* gene for the control and high BCM samples showed a similar species richness for the libraries, however, the populations that make up the libraries were significantly altered between samples (libshuff *p* < 0.001). Sequences with a homoacetogen similarity score (HSS) >80 were found to lie in the acetogenic bacteria clustering of the FTHFS phylogenetic tree. From this region the predominant FTHFS OTU’s for the control sample were positioned close to *Ruminococcus obeum*, while for BCM the most predominate OTU grouped closest to *Clostridium magnum*. Four OTU’s comprising 43% of the sequences in the BCM sample were associated with the mixotrophic acetogen *Sporomusa* spp., while only a single representative from the control library was observed in this group.

Acetyl CoA synthase libraries were also generated using recently designed primers that exclude sulfate reducing bacteria and archaeal *acsB* genes (Gagen et al., 2010). A greater diversity of sequences was observed from the control samples and the composition of the BCM sample tended to be a subset of the control sample (libshuff *p* = 0.33), but the control sample was different from the high BCM due to its increased diversity (libshuff *p* < 0.003). The most abundant OTU was the same for both samples being associated closest to *Acetitomaculum ruminis*, while, the rank abundance of the remaining OTU’s was different between the samples.

### Metagenomic Data Analysis of Goat Microbiome

Genomic DNA from all animals was pooled together based on sampling period and subjected to 454 titanium shot gun sequencing for the control and high BCM samples. Replicated sequences due to construction of the 454 sequencing libraries were identified and accounted for 19% of the control and 22% of the high BCM metagenomic datasets. Assembly of metagenomic reads produced over 6500 contigs greater than 500 bp for the control sample, while in excess of 9000 contigs were generated for the High BCM metagenome. The largest assembled contig for the BCM library was close to 40 kb in length, while the largest contig for the control was just over 6.4 kb. Annotation of the 40 kb high BCM contig identified the coding sequences belonging to previously described genes from *Prevotella* species, while the largest contig for the control sample could only be accurately classified to the Firmicutes phylum level.

**FIGURE 3:**
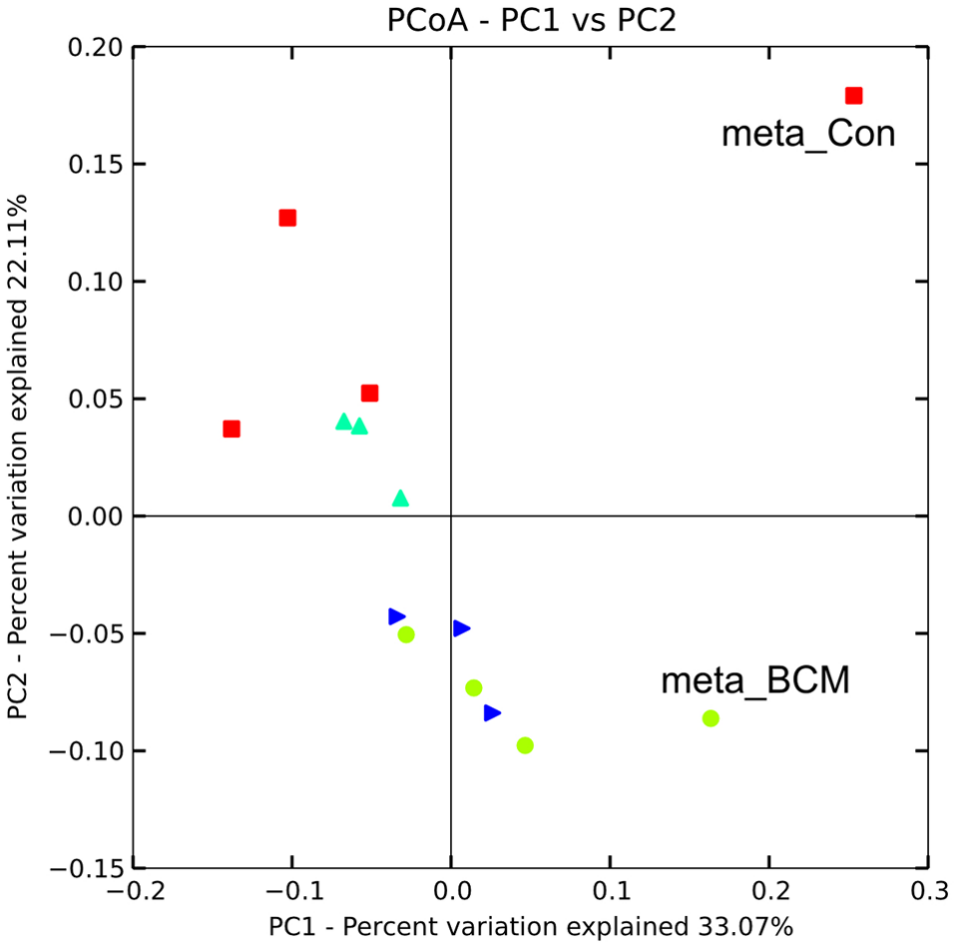
**Weighted Unifrac diversity principal coordinate analysis of 16S rDNA amplicon generated rumen microbiomes and 16S rDNA sequences extracted from metagenomic sequencing for animals on varying levels of BCM for control (red square), low dose (turquoise triangle), mid dose (blue right triangle), high dose (green circle).** Point’s labeled meta_con and meta_BCM indicate 16S rDNA sequences obtained from metagenomic sequencing of pooled animal samples for the control and high BCM period, respectively.

Hidden Markov models were used to identify sequences containing 16S rDNA data, resulting in 435 and 700 sequences for the control and high BCM samples, respectively. Clustering of the metagenomic 16S sequences in association with the 16S amplicon data to a Greengenes 97% reference set captured 402 and 619 of the metagenomic reads for the control and high BCM samples. Similar to the amplicon data, the control and high BCM samples were divergent in their composition (**Figure 3**). The control metagenomic 16S rDNA data, although separated from the high BCM meta 16S rDNA data did not group closely with the control 16S rDNA amplicon data, while the high BCM meta 16S rDNA data was more closely related to the high BCM amplicon data (**Figure 3**). The rank abundance of phyla for the control were in agreement for the most abundant populations, those being from the Bacteroidetes and Firmicutes, however, the 16S rDNA sequences from the metagenomic data also detected high levels of Verrucomicrobia and Actinobacteria which where only observed as a minor proportion of the amplicon sequences. The high BCM samples were in closer agreement with each other and had a similar rank abundance, but again the amplicon data only detected a small contribution of Verrucomicrobia compared to the metagenomic 16S rDNA data (Supplementary Figure S6).

**FIGURE 4:**
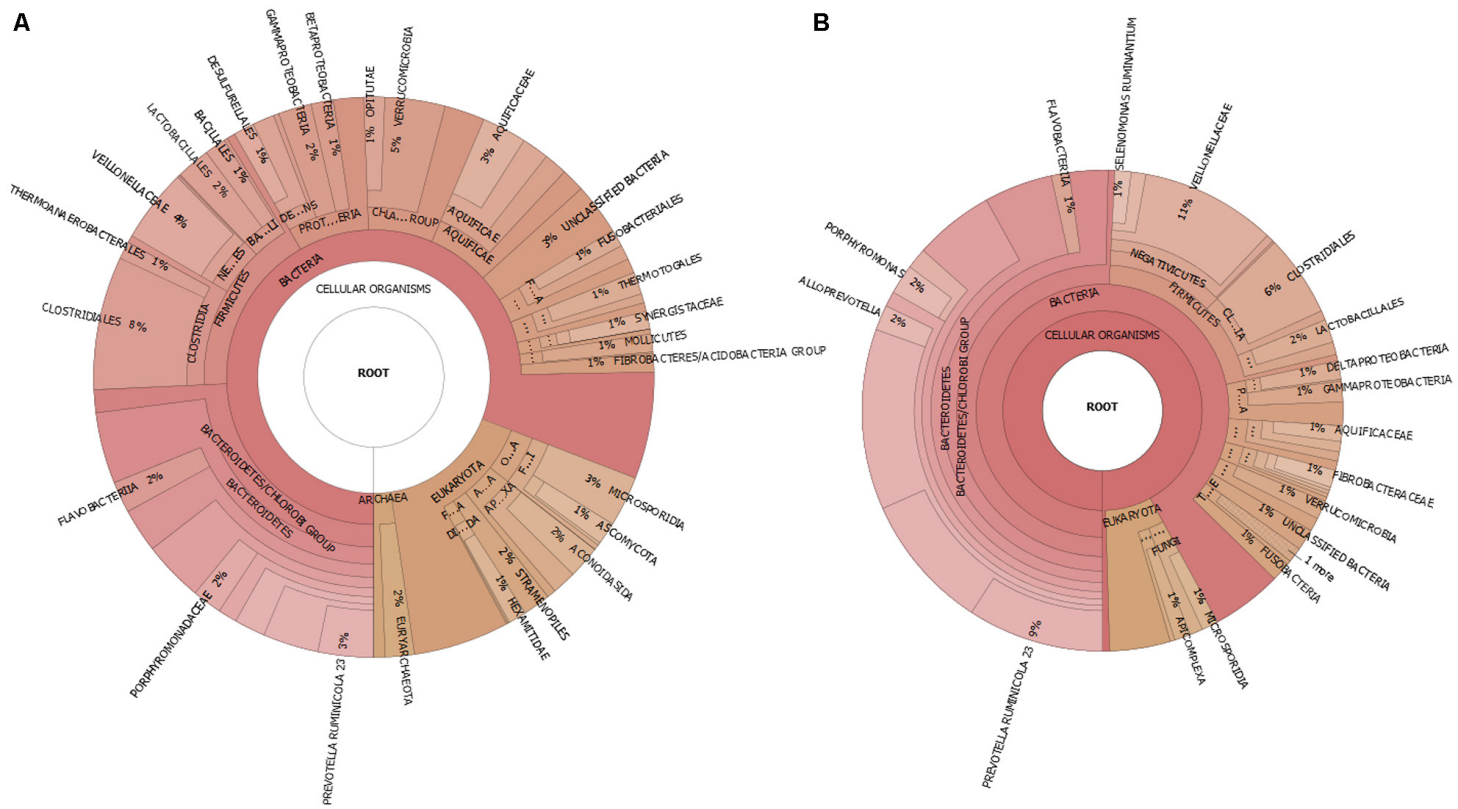
**Taxonomic visualization of goat rumen microbiome for (A) control and (B) high BCM metagenomics sequence data**.

Further analysis of the microbial community using PhyloSift based on the single-copy 37 “elite” gene families (Darling et al., 2014), also concluded a similar rank abundance and variance for the major phyla between the control and high BCM samples (**Figure 4**). Although the absolute values were not similar, the changes for the abundance of the phyla due to BCM was consistent with increases in Bacteroidetes and decreases in Proteobacteria and no real change to the Firmicutes. Archaeal sequences were only identified within the control metagenomic sample, as were sequences associated with Synergistetes and Tenericutes phyla. These were found to be reduced in abundance in the high BCM amplicon datasets.

Phylogentic classification of the assembled and unassembled reads using PhyloPythiaS assigned ∼52% of the data to the phylum level or lower. RAIphy classification assigned 96–94% of the reads to the phylum level or below for the high BCM and control data respectively. The observed number of phyla was greater in the metagenomic binning methods than for the 16S rDNA and PhyloSift approaches and included reads assigned to phyla that are not expected to be present in the rumen environment. These were predominantly thermophilic phyla and only represented a small proportion of the taxonomic assignment. For both metagenomic samples there was a high proportion of reads assigned to the Bacteroidetes and Firmicutes as was observed for the 16S rDNA and PhyloSift analysis.

**FIGURE 5:**
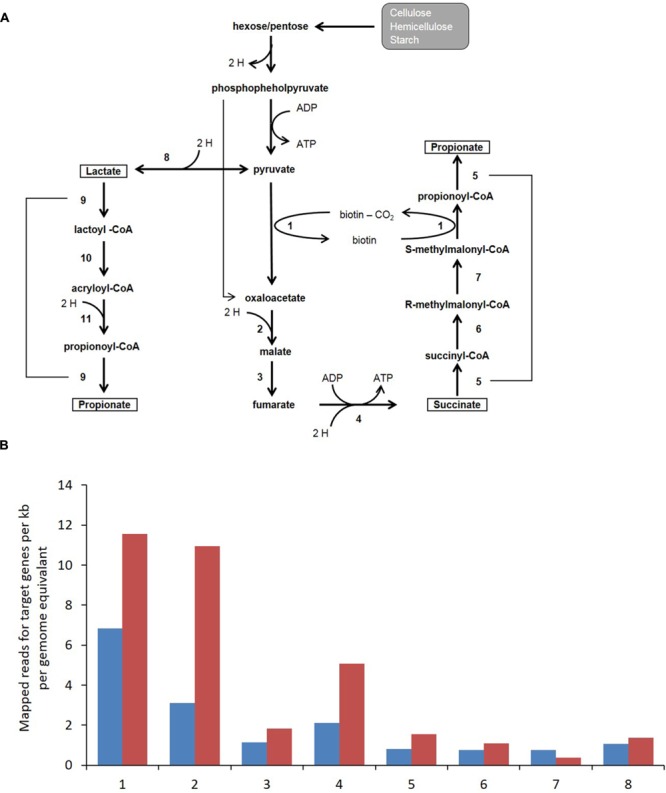
**(A)** Microbial fermentation pathway for propionate production which consumes H_2_ via the randomizing (succinate) or non-randomizing (acrylate) pathways. Numbers refer to enzymatic conversion catalyzed by 1, transcarboxylase (pyruvate carboxylase, methylmalonyl-CoA decarboxylase or methylmaloynyl-CoA carboxytransferase); 2, malate dehydrogenase; 3, fumarase; 4, fumarate reductase; 5/9, propionyl-CoA transferase; 6, methylmalonyl-CoA mutase; 7, methylmalonyl-CoA epimerase; 8, lactate dehydrogenase; 10, lactoyl-CoA dehydratase; 11, acryloyl-CoA reductase. **(B)** Bar charts indicates metagenomic reads assigned to a given target gene per kb per genome equivalent for control (blue) and High BCM (red). Numbers on the x axis correspond to the same enzymatic steps as indicated in **(A)**.

Annotation and functional assignment of coding genes was performed at the MG-RAST server (http://metagenomics.anl.gov/). Approximately 380,000 and 350,000 protein coding regions were identified for the control and high BCM samples of which 36.5 and 46.5% could be assigned a known function. Enzymes involved in the biochemical pathway for the production of propionate in the rumen utilizing the randomizing (succinate) pathway were identified within the metagenomic data (**Figure 5** and Supplementary Figure S7). There was an absence of lactyl-CoA dehydratase and acryloyl-CoA-reducatse reads for the non-randomizing (acrylate) pathway from either metagenomic dataset. The abundance of reads associated with the enzymes of the randomizing pathway from pyruvate, was increased approximately twofold on average for the high BCM rumen compared to the control sample. Representatives from six families contained the entire pathway for the assignment of the genes involved in the decarboxylation of succinate to propionate: *Chlorobiaceae, Desulfovibrionaceae, Fibrobacteraceae, Hyphomicrobiaceae, Porphyromonadaceae, Prevotellaceae*. The *Prevotellaceae* group contained the highest representation of genes for the propionate pathway. A further ten families contributing at a lower abundance were identified to contain all but one enzyme for the succinate to propionate pathway and included *Acidobacteriaceae, Bacteroidaceae, Bifidobacteriaceae, Clostridiaceae, Coriobacteriaceae, Corynebacteriaceae, Geobacteraceae, Rikenellaceae*, and *Veillonellaceae.* Reads assigned to *Propionibacteriaceae* showed an incomplete pathway with no hits to either fumarase or methylmalonyl-CoA epimerase genes and were only observed in the BCM sample at low abundance.

**FIGURE 6:**
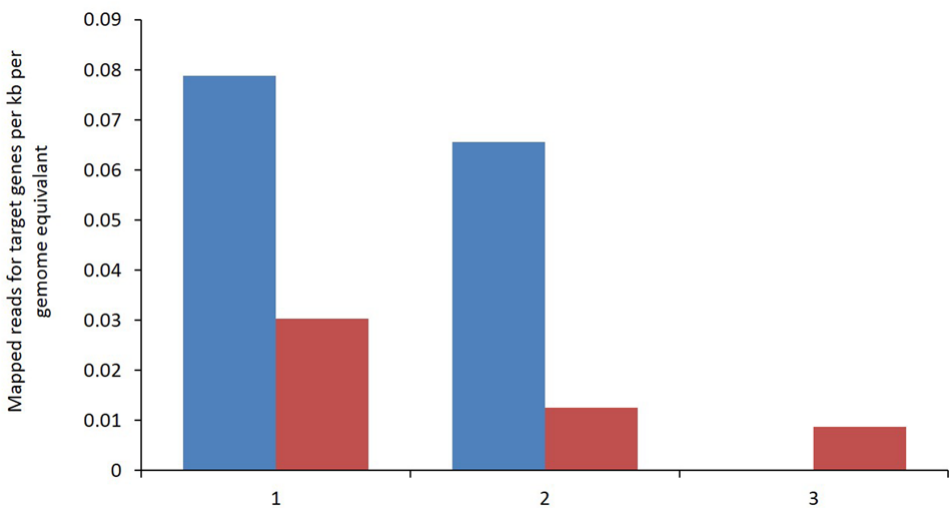
**Metagenomic reads assigned to a given target gene per kb per genome equivalent for control (blue) and High BCM (red).** (1) CO dehydrogenase/acetyl-CoA synthase complex, (2) methyltetrahydrofolate:corrinoid/iron–sulfur protein and (3) corrinoid/iron–sulfur protein.

Enzymes involved in the reductive acetogenic pathway that possibly provide the best evidence for reductive acetogenic bacteria including the carbon monoxide dehydrogenase/Acetyl CoA synthase (CODH/ACS), corrinoid/Fe–S:methyl-transferase and a corrinoid/Fe–S protein were identified. Only limited sequence data was mapped for these key enzymes from both metagenomic libraries emphasizing the relatively low abundance of bacteria capable of reductive acetogenesis (**Figure 6**). All genes were assigned to the clostridiales class of bacteria. The data, although limited, shows a reduction in the identification of these key enzymes in the BCM treated rumen.

## Discussion

The administration of BCM-CD at 5 g/100 kg live weight lead to a 91% reduction in methane production with a concurrent increase in ruminal H_2_ levels with no apparent negative effect on dry mater intake (Mitsumori et al., 2012). Initial investigations into the microbial population’s shifts due to the introduction of BCM identified reductions in the methanogen population, shifts in fibrolytic populations and the increase in *Prevotella* spp. (Mitsumori et al., 2012). Further analysis presented here, using extensive sequencing of the rumen microbiome through targeted 16S rDNA amplicons and metagenomic sequencing were in agreement with these previous results and provide greater insight into the complexity of the microbiome shifts.

The increasing concentration of BCM administered to goats altered the microbial diversity through a reduction in the richness and overall diversity of the bacterial species observed. At the highest level of BCM the rumen microbiomes showed the least divergence. The alteration to the rumen environment with increased levels of H_2_ affected the various goat rumens to an equivalent extent, thus overcoming the original animal to animal variance observed in the control and lower doses of BCM. Animal 418 exhibited a higher level of microbial richness for alpha diversity measures and was more divergent from the other two animals as measured through beta diversity metrics (unifrac analysis), but this was reduced at the highest level.

Many bacterial OTUs were positively associated with the increasing concentration of BCM in the rumen, with the most dominant species being two *Prevotella* spp. that were previously identified through DGGE and qPCR analysis to be closely related to *Prevotella ruminicola* (Mitsumori et al., 2012). They also exhibited some of the highest correlation values for the increasing BCM concentration, indicating a true dose response. The increase in these OTUs was primarily responsible for the overall decrease in alpha diversity observed in the high BCM animals. In addition seven OTUs classified in the S*elenomonas* genus were also positively associated with the increase in BCM. Both *P. ruminocola* and *Selenomonas ruminatium* are known propionate producers (Paynter and Elsden, 1970; Strobel, 1992). Co-culturing of *S. ruminatium* with the fiber degrading succinate producer *F. succinogenes* on cellulose illustrated how interspecies interactions are possible in the rumen with the non-celluloltyic *S. ruminantium* decarboxylation of succinate to propionate (Scheifinger and Wolin, 1973). In line with the observed increase in *S. ruminantium* an increase in *F. succinogenes* was also noted in the 16S rDNA sequence data and quantified previously with qPCR. *F. succinogenes* is not negatively affected by increases in H_2_ concentration like the *Ruminococcus* species (Wolin et al., 1997). Interestingly, both the qPCR data and the sequencing data show the highest level for *F. succinogenes* at the mid BCM dose with a slight reduction at the highest BCM level. This plateau in abundance for *F. succinogenes* explains the moderate association to the changing concentration of BCM.

Bacteria that were negatively correlated by the increase in BCM and subsequent increases in H_2_ concentration within the rumen were dominated by species from two groups; those from the Dethiosulfovibrionacaea family within the Synergistes phylum and those from the Victivallaceae family from the Lentisphaerae phylum. *Victivallis* spp. OTUs were found to be distantly related to *Victivallis vadensis*, a human fecal isolate that can grow on cellobiose or glucose producing acetate, ethanol, H_2_ and bicarbonate. However, like most H_2_ producers, when grown syntrophically with a methanogen species, *V. vandensis* converted glucose exclusively to acetate and H_2_ (Zoetendal et al., 2003). A feature similar was observed for the fibrolytic species *Ruminococcus flavefaciens* (Wolin et al., 1997). Likewise, studies of members of the Dethiosulfovibrionacaea family revealed stimulation of growth through removal of high H_2_ concentrations (Surkov et al., 2001). These groups of bacteria would seem to be consistently down regulated due to the higher H_2_ concentrations being generated in the BCM treated rumens.

Metagenomic sequencing of the control and high BCM goat rumen microbial communities provided evidence of a reduction in reads assigned to archaeal genomes in the BCM sample and supports the previous findings of the reduction of measured methane and methanogen numbers (Mitsumori et al., 2012). In addition a decrease in eukaryotic reads associated with fungal and protozoal genomes in the BCM sample likely reflects the negative impact of the higher H_2_ concentrations in the treated rumen (Bauchop and Mountfort, 1981; Ushida and Jouany, 1996). Increased H_2_ concentrations are known to alter the fermentative end products of H_2_ producing bacteria, fungi, and protozoa toward less energy yielding reduced compounds like lactate, ethanol, and butanol (Wolin, 1974). The presence of methanogen species allows for the reduction of the H_2_ partial pressure and the favorable conditions for the reoxidation of nicotinamide adenine dinucleotide (NADH) and redirection of the reducing equivalents toward more energy yielding products like acetate for the fermentative species (Wolin, 1974; Bauchop and Mountfort, 1981; Hillman et al., 1988; Teunissen et al., 1992; Ushida et al., 1997; Wolin et al., 1997). Although previous observations of these rumen samples confirmed a reduction in fungal numbers through qPCR, the protozoal numbers as determined by microscopic counts were not different (Mitsumori et al., 2012). As the current genomic databases are limited for representatives from rumen protozoal and anaerobic fungal this is likely to cause inaccuracies in these assignments. Further work to quantify the protozoal numbers using a qPCR assay should be undertaken to confirm this reduction in their contributions to the rumen microbiome. The reduction in eukaryotic reads lead to a concurrent increase in reads being assigned to bacterial origin and reflects the constraining of the diversity due to the administration of BCM. Moreover a shift in the GC% for the sequences was observed in the BCM sample with an increase in reads with a GC% range of 40–70%. This would be in agreement with the observed increase in Bacteroidetes numbers, especially *Prevotella* groups in the 16S OTU data. Most phylum level classification of the metagenomics reads was also in agreement with the OTU data for the samples not only in abundance levels, but similar in the changes to the percentage of the data set represented between the samples.

Phylogenetic binning and functional assignment of metagenomics reads resulted in two major genera, *Prevotella* and *Selenomonas*, being confidentially assigned all enzymes involved in the randomizing succinate pathway. Both of these groups were also assigned lactate dehydrogenase activity, which converts pyruvate to lactate for use in the non-randomizing acrylate pathway (Prins and Van Der Meer, 1976). Neither lactyl-CoA dehydratase or acryloyl-CoA reductase were found in either of the metagenomic datasets, indicating that the non-randomizing acrylate pathway is not the major route for the production of propionate in the rumen or at least not contained within the abundant bacterial groups. As lactate dehydrogenase is a reversible reaction, it is capable of converting lactate to pyruvate for use in the randomizing succinate pathway (Paynter and Elsden, 1970). Based on these data, the major shift in bacterial population abundance in response to BCM can be attributed to *Prevotella* and *Selenomonas* sp. utilizing pathways for propionate production which consume H_2_ via the randomizing (succinate) pathway through the fermentation of sugars and lactate (Bryant et al., 1958; Strobel, 1992; Purushe et al., 2010). It is likely that these pathways were the primary routes for consumption of H_2_, which accumulated as a consequence of reduced methanogenesis. The development of qPCR primers to monitor two of these *Prevotella* clusters (Mitsumori et al., 2012) confirmed the observations of the DGGE analysis and 16S OTU data, in that these were dominant bacterial populations that increased in abundance at high BCM dosing levels. However, other bacteria such as *Megasphaera elsdenii*, *S. ruminantium*, *Succinimonas amylolytica*, *Propionibacterium acnes* and *Veillonella parvula*, were observed to change and may also be involved (Hungate, 1966; Marounek et al., 1989; Stewart et al., 1997; Wolin et al., 1997). Further studies involving deeper sequencing and tracer experiments with C-labeled sugars and lactate are required to determine the relative contribution of these pathways as the rumen microbiota adapts to high H_2_ concentration in the rumen.

With a decrease in methanogenesis and subsequent increase in ruminal H_2_ concentrations, reductive acetogens are able to compete successfully with methanogens for H_2_ within the rumen (Ungerfeld and Kohn, 2006). In this regard shifts in bacterial populations carrying key functional genes from the reductive acetogenesis pathway such as *acsB* and *fhs* should identify changes to these populations. The administration of high doses of BCM-CD (5 g/100 kg live weight) significantly altered and restricted the diversity of the FTHFS sequences. The restriction in diversity is in contrast to a previously reported study for cattle supplemented with BCM-CD at a dose rate of 1 g/100 kg live weight, which produced marked changes in the populations that carry the *fhs* gene but produced an increase in diversity (Mitsumori et al., 2014). The restriction in diversity in the goats is likely a reflection of the higher doses of BCM and reveals a negative impact on certain acetogenic populations. BCM is a halogenated methane analog that’s mode of action is through reacting with reduced vitamin B_12_ and inhibiting the cobamide-dependent methyl transferase step of methanogenesis (Wood et al., 1968; Chalupa, 1977). The B_12_ dependent methyl transferases also play an important role in one carbon metabolism in acetogenic bacteria (Banerjee and Ragsdale, 2003), and therefore BCM may have an effect on reductive acetogenesis, which has not been investigated. One of the groups that was prevalent in the BCM treated goats was associated closely to *Sporomusa ovata*. *S. ovata* is unique in its requirement for phenolyl cobamides as opposed to the more common benzimidazolyl cobamides associated with acetogenic and methanogenic methyl transferases (Stupperich et al., 1988; Kaster et al., 2011; Mok and Taga, 2013). This preference for phenolyl ligand cobamides and the ability of *Sporomusa* species to grow more productively on organic substrates such as methanol and lactate in conjunction with H_2_ oxidation to produce acetate gives these species an advantage over purely reductive acetogens (Breznak and Blum, 1991). Experiments to investigate the effect that BCM may have on acetogenic bacterial isolates need to be performed to ascertain any negative mode of action.

Existing tools targeting the FTHFS sequence as a marker for reductive acetogenesis are compromised by lack of specificity due to the involvement of FTHFS in other pathways (Drake et al., 2008; Pierce et al., 2008). ACS is unique to the acetyl-CoA pathway and is an excellent marker gene for detecting acetogenic bacteria. However, as this pathway is also used by some methanogens and sulfur reducing species for generation of cell carbon and/or acetoclastic growth some caution in interpretation is still required (Ragsdale, 1991). A recently designed primer set that excludes, or at least minimizes these non-acetogenic populations has proven to target acetogenic populations from gut environments (Gagen et al., 2010). Similarly, the only ACS sequences recovered from the goat rumen using this primer set showed affiliation to groups between the Lachnospiraceae and Clostridiaceae acetogen families. Within the BCM treated animals the majority of the ACS sequences were associated with *A. ruminis*. This grouping was not strictly identified using the FTHFS gene analysis possibly due to the limitations of the FTHFS primers to amplify the gene from these species (Henderson et al., 2010). However, the dominant FTHFS OTU closely located with *C. magnum* is in the same position in the tree that *A. ruminis* is located (Gagen et al., 2010). It is likely that this group of bacteria are the dominant acteogens in the BCM treated goat rumen. In addition, the lack of congruency for the grouping of FTHFS genes associated with the *Sporomusa* species and ACS sequences can be explained in that these grouping are not supported by bootstrapping for these species when comparing tree placements between FTHFS and ACS libraries and is therefore likely to be placed inaccurately within the ACS tree (Gagen et al., 2010).

## Conclusion

The dose-dependent inhibitory effect of BCM on methanogens in the goat rumen with measured reductions in methane and increases in H_2_ has both directly and indirectly effected the rumen microbiome. Rumen fermentation end products were mostly unchanged with respect to the addition of BCM, but significant increases in propionate and iso-valerate were detected at the mid and high doses. In depth microbial ecology and metagenomic analysis allowed high-resolution observations into the changes in rumen microbial populations with respect to abundance and presumed level of metabolic capacity within the system. Metagenomic analysis was dominated by genomic content from *Prevotella* and *Selenomonas* species and showed potential to produce propionate through the randomizing (succinate) pathway. Reductive acetogenic populations were also affected significantly by the changes in the rumen environment. Both markers gene studies and metagenomics data suggest that they provide minor contributions to the redirection of H_2_ in BCM treated animals.

## Conflict of Interest Statement

The authors declare that the research was conducted in the absence of any commercial or financial relationships that could be construed as a potential conflict of interest.
