# Supplementary material for: Metagenomic analysis of the rumen microbial community following inhibition of methane formation by a halogenated methane analog
Source: Front Microbiol. 2015 Oct 13;6:1087. doi: 10.3389/fmicb.2015.01087 (PMC4602129; doi:10.3389/fmicb.2015.01087)
Supplement: Supplementary file 1 [file Data_Sheet_1.DOCX]

***Supplementary Material***

**Metagenomic analysis of the rumen microbial community following inhibition of methane formation by a halogenated methane analogue**

**Stuart E. Denman^1^*, Gonzalo Martinez Fernandez^1^, Takumi Shinkai^2^, Makoto Mitsumori^2^ and Christopher S. McSweeney^1^**

^1^ Queensland Bioscience Precinct, Agriculture Flagship, CSIRO, St Lucia, QLD, Australia

^2^National Institute of Livestock and Grassland Science, Tsukuba, Ibaraki, Japan

*** Correspondence:** Stuart E. Denman, Queensland Bioscience Precinct, Agriculture Flagship, CSIRO, 306 Carmody Road, St Lucia, QLD, 4067, Australia. Email:Stuart.denman@csiro.au

1. **Supplementary Figures**


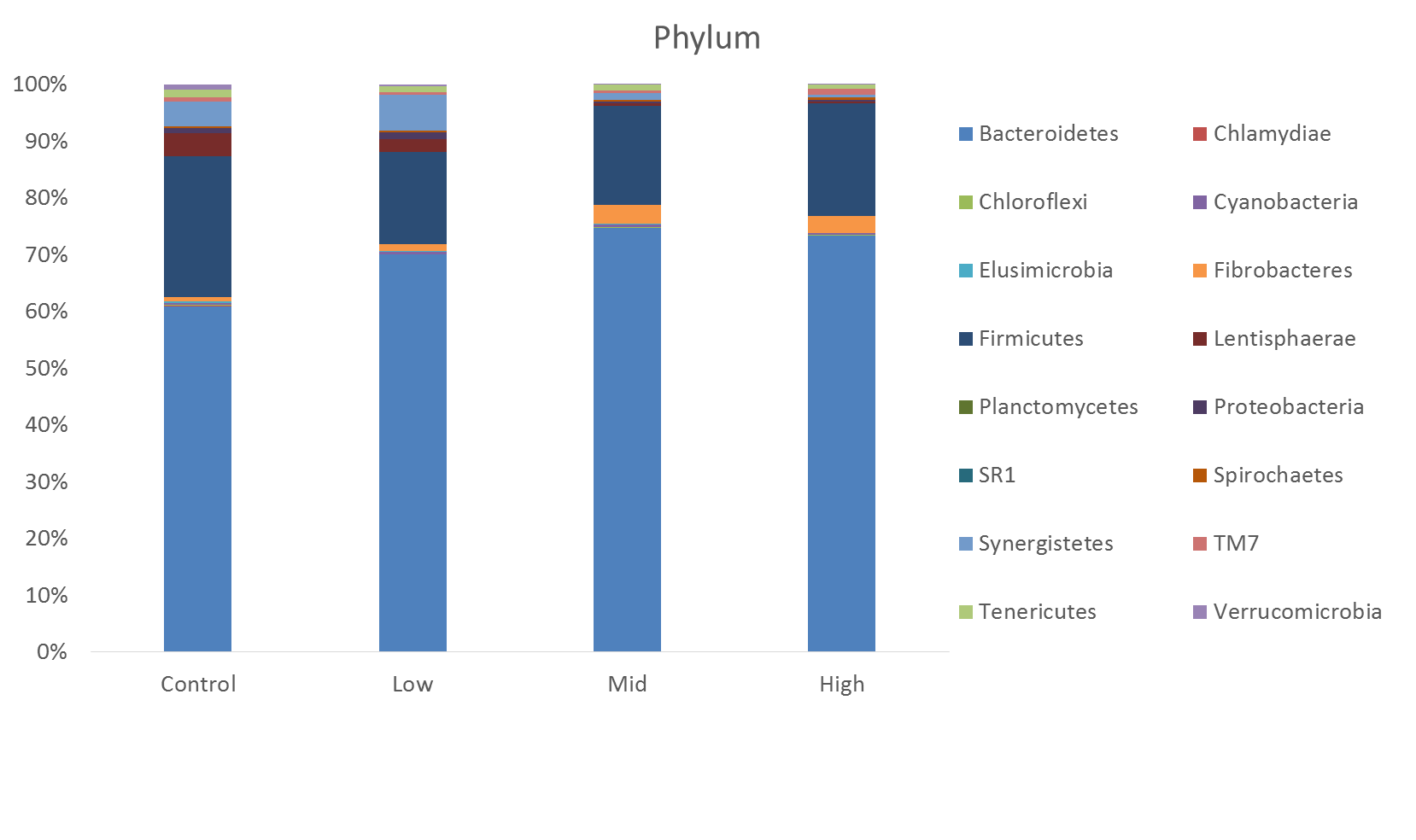
**Supplemantary Figure 1.** Phylogentic taxonomic assignment of 16S rDNA reads at the phylum level for control, low, mid and high doses of BCM.


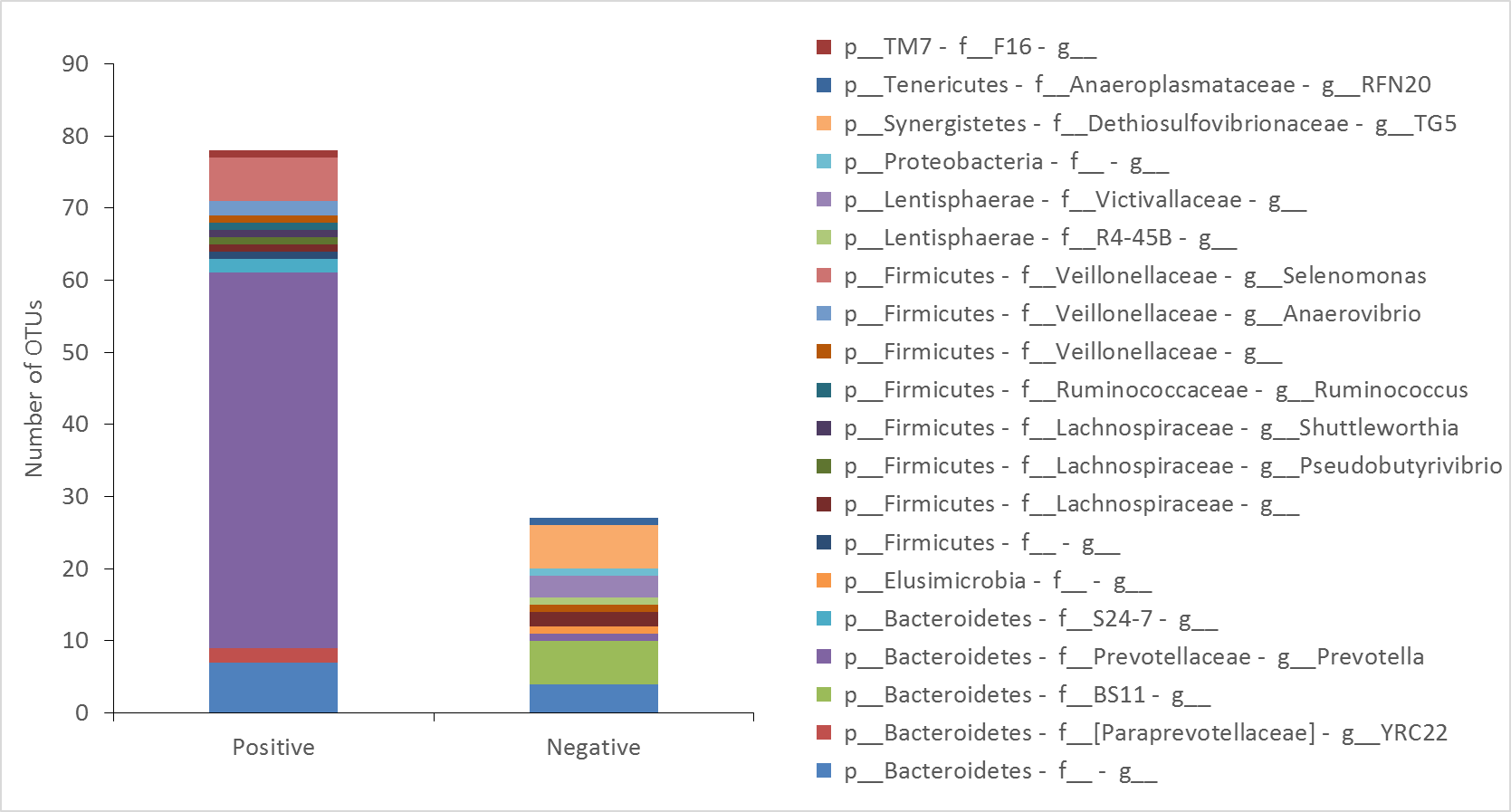


**Supplementary Figure 2**. Number of OTUs positively (r > 0.6) or negatively ( r < -0.6) associated to changes in BCM concentration administered into the rumen.


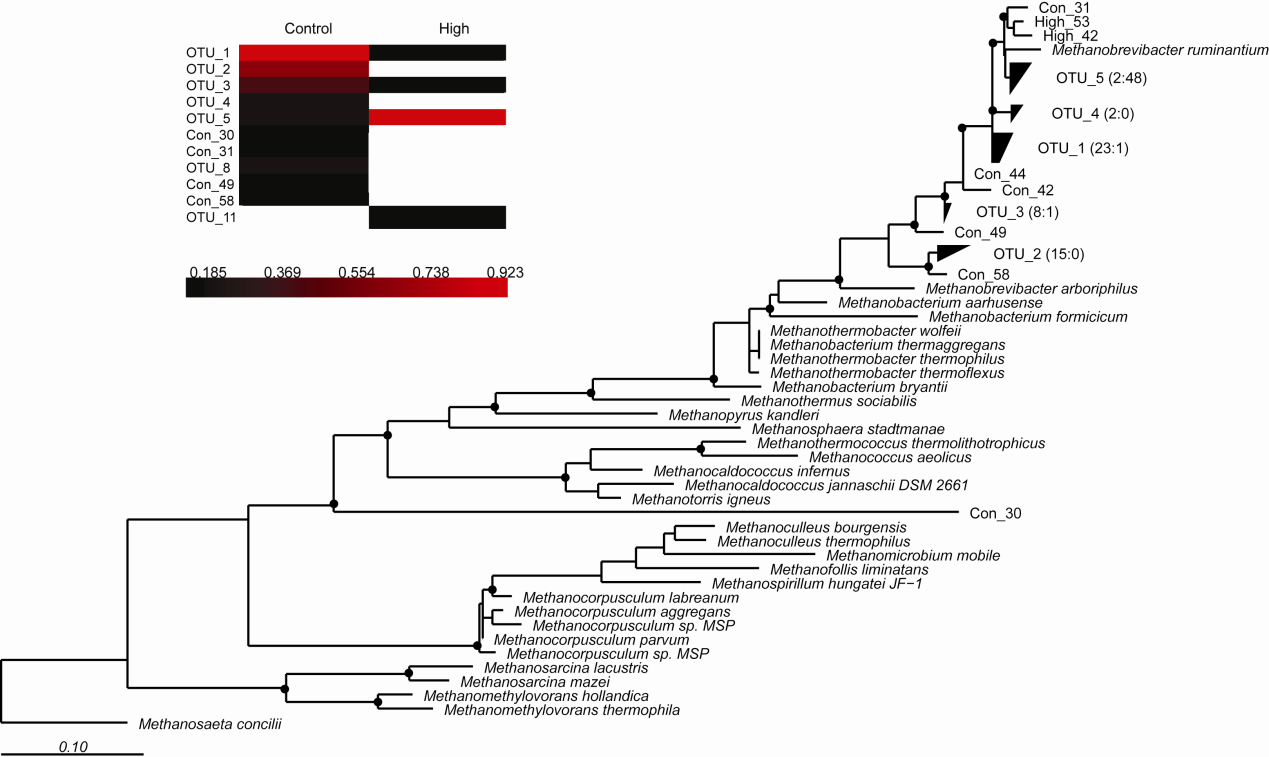


## Supplementary Figure 3. Phylogenetic placement of mcrA clones (maximum likelihood). Numbers in brackets indicate the contribution to the OTU from control and BCM high respectively. Black circles at nodes represent a bootstrap value > 50%. Insert of heatmap showing abundance of sequences in a given OTU.


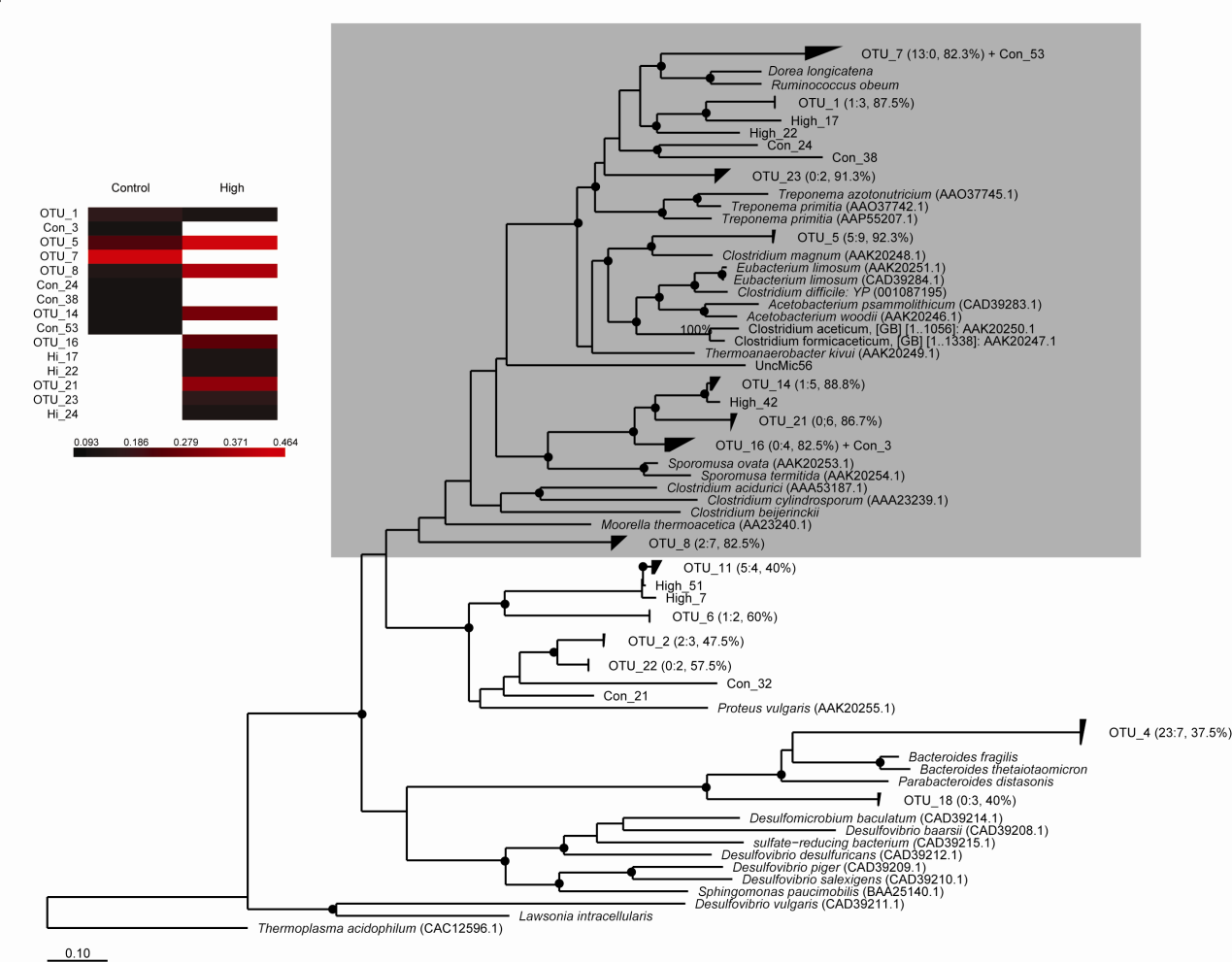


**Supplementary Figure 4.**Phylogenetic placement of FTHFS clones (maximum likelihood). Numbers in brackets indicate the contribution to the OTU from control and BCM high respectively. Black circles at nodes represent a bootstrap value > 50%. Shaded region indicates acetogenic bacteria clusters. Insert of heatmap showing log2 abundance of sequences in a given OTU for sequences with a HSS > 80%.


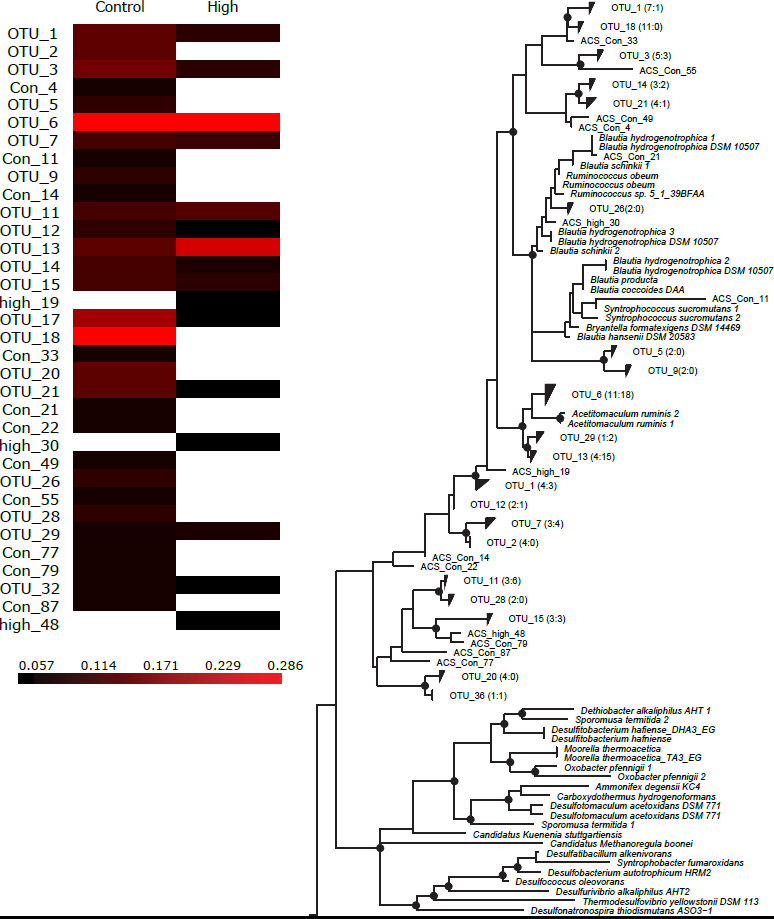


**Supplementary Figure 5.** Phylogenetic placement of ACS clones (maximum likelihood). Numbers in brackets indicate the contribution to the OTU from control and BCM high respectively. Black circles at nodes represent a bootstrap value > 50%. Insert of heatmap showing log2 abundance of sequences in a given OTU.


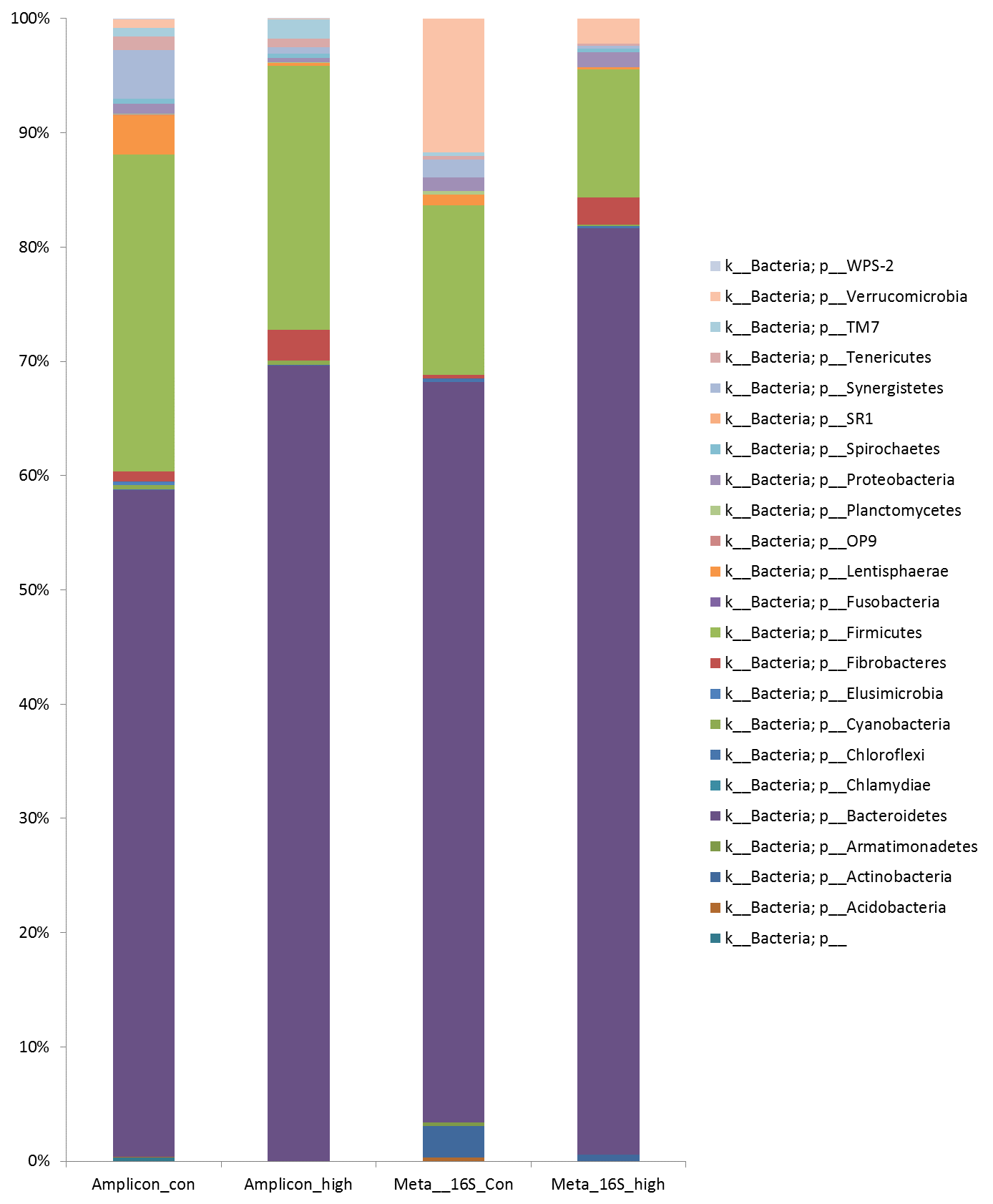


**Supplementary Figure 6.** Phylogentic taxonomic assignment of 16S rDNA reads at the phylum level for amplicon generated for control and high BCM and 16S rDNA sequences identified from control and high BCM metagenomic sequencing.


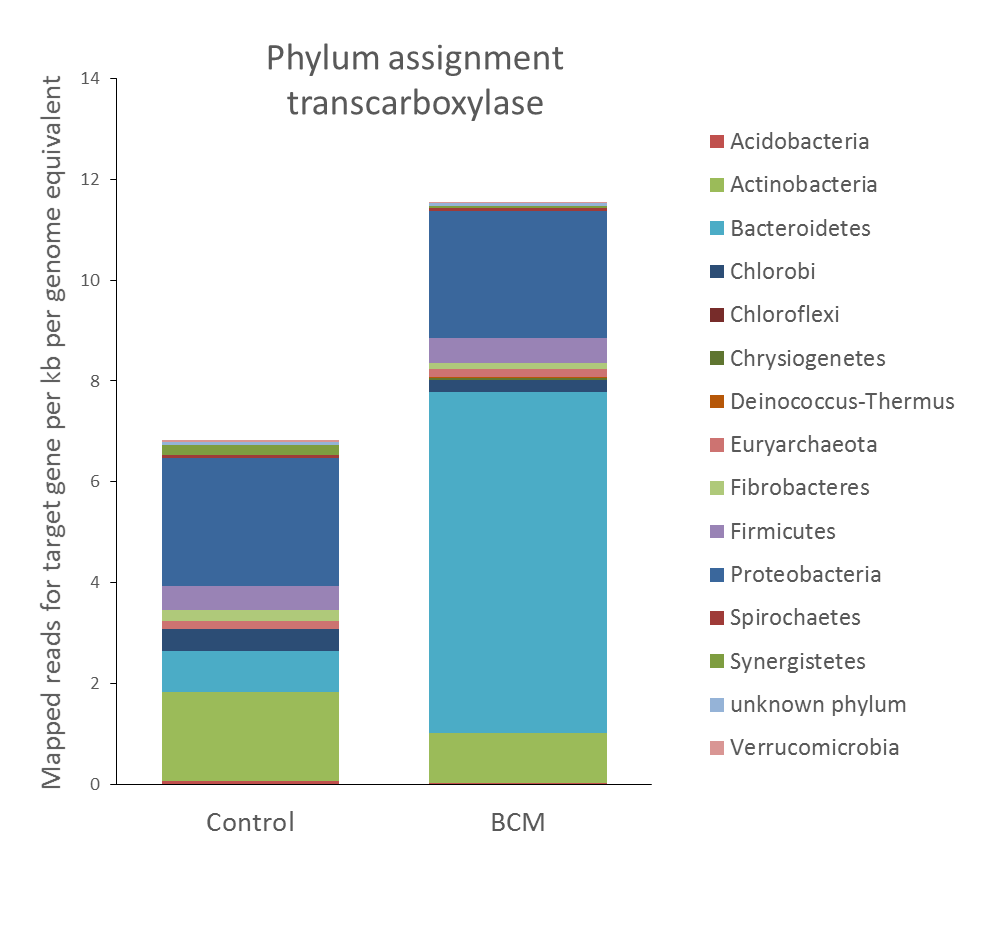


**Supplementary Figure 7**. Phylum level classification for reads assigend to a) transcarboxylase pathway


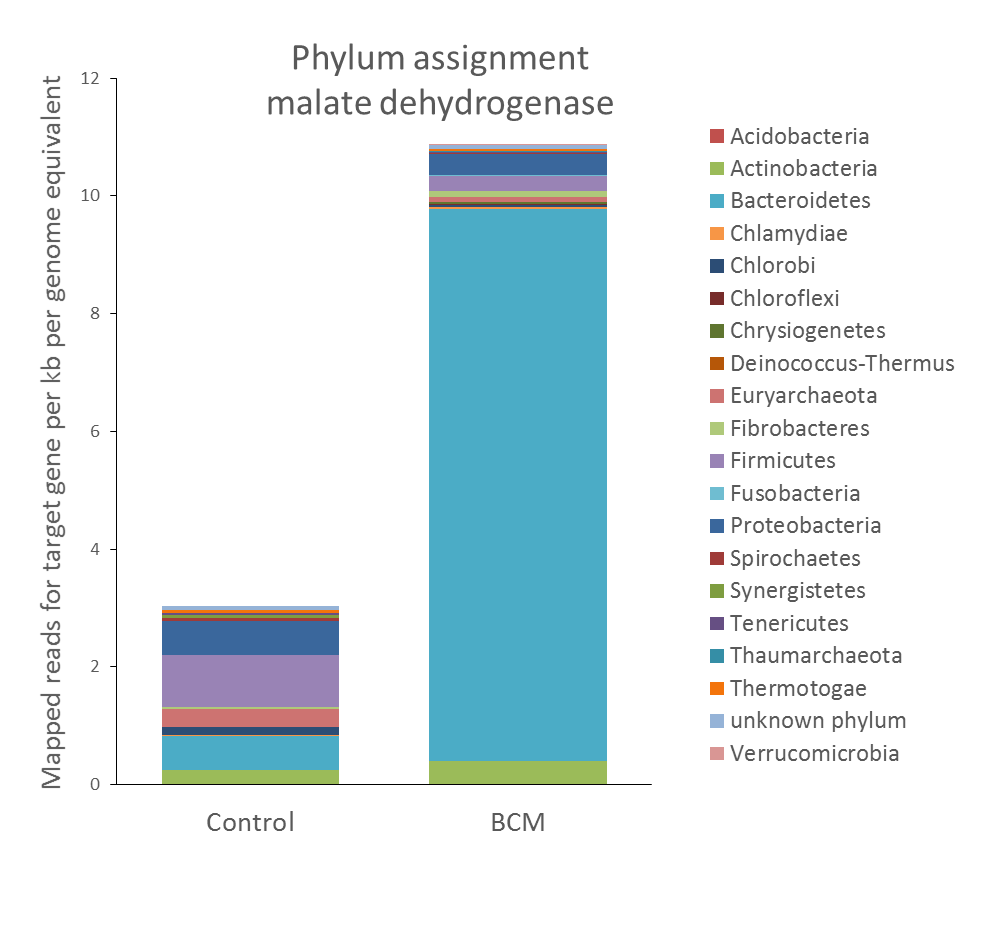


**Supplementary Figure 7**. Phylum level classification for reads assigend to b) malate dehydrogenase pathway


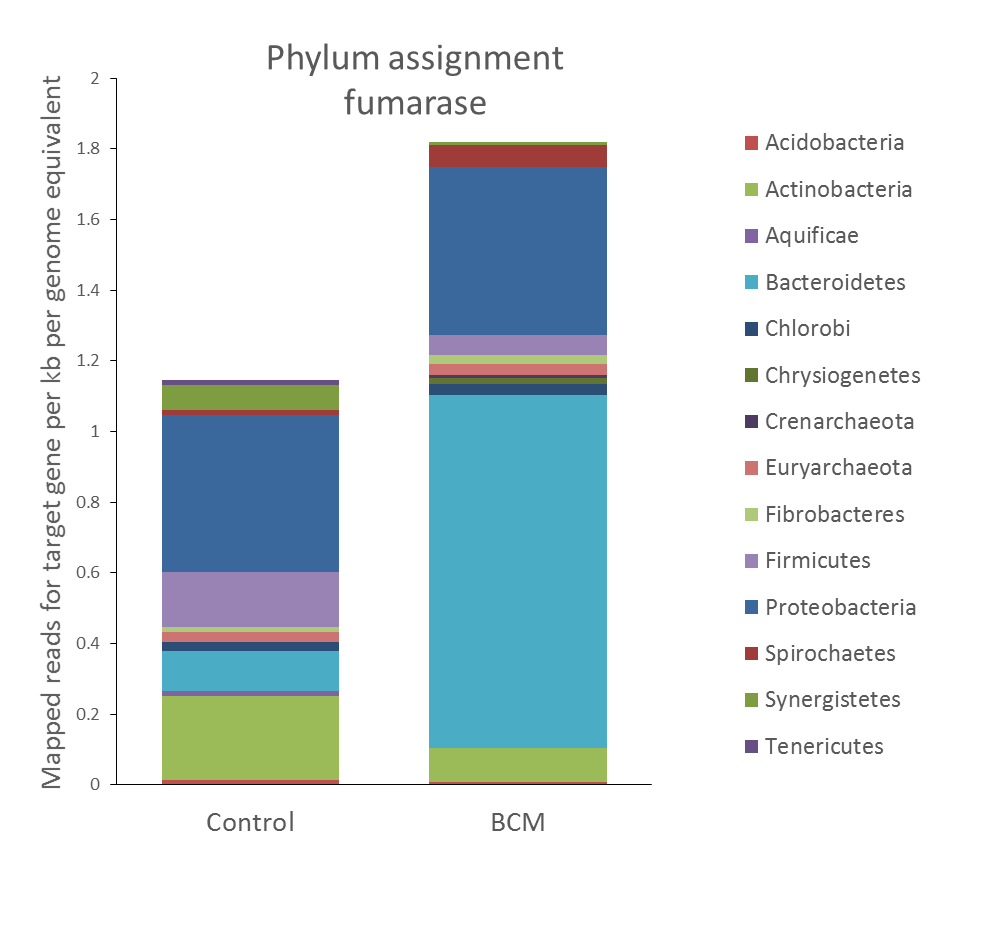


**Supplementary Figure 7**. Phylum level classification for reads assigend to c) fumarase pathway


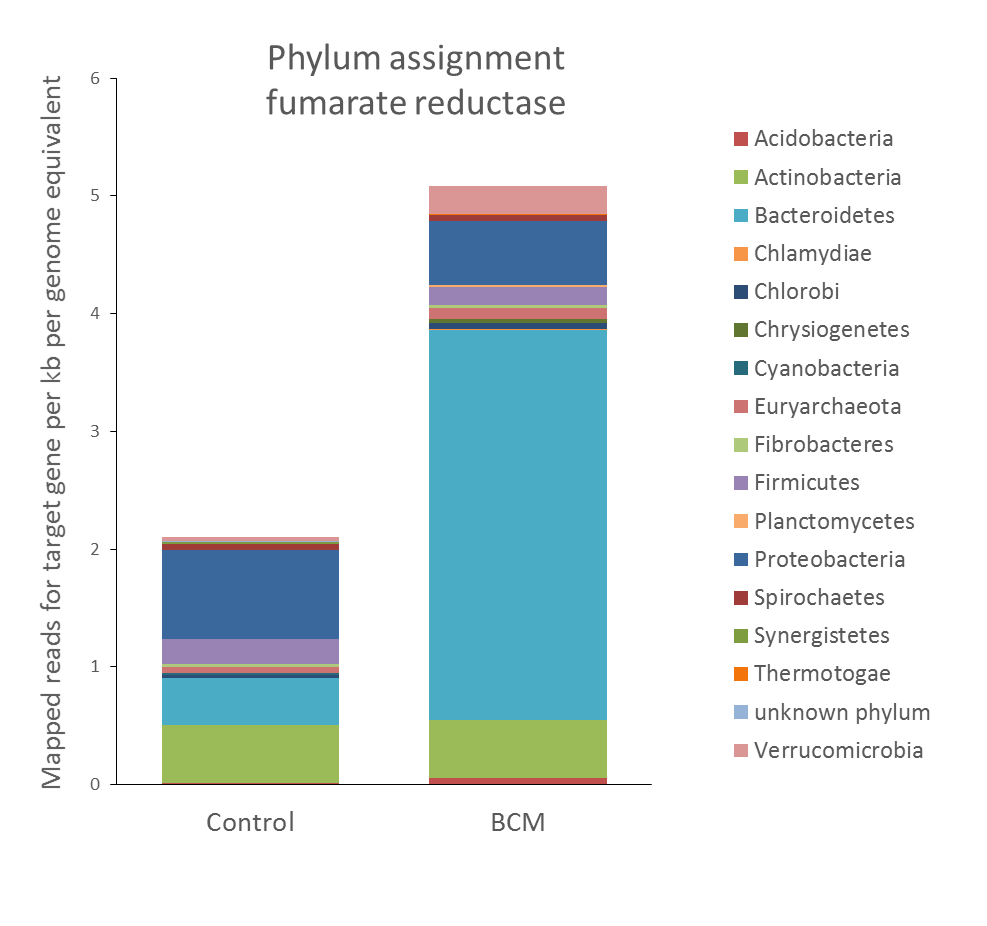


**Supplementary Figure 7**. Phylum level classification for reads assigend to d) fumarate reductase pathway


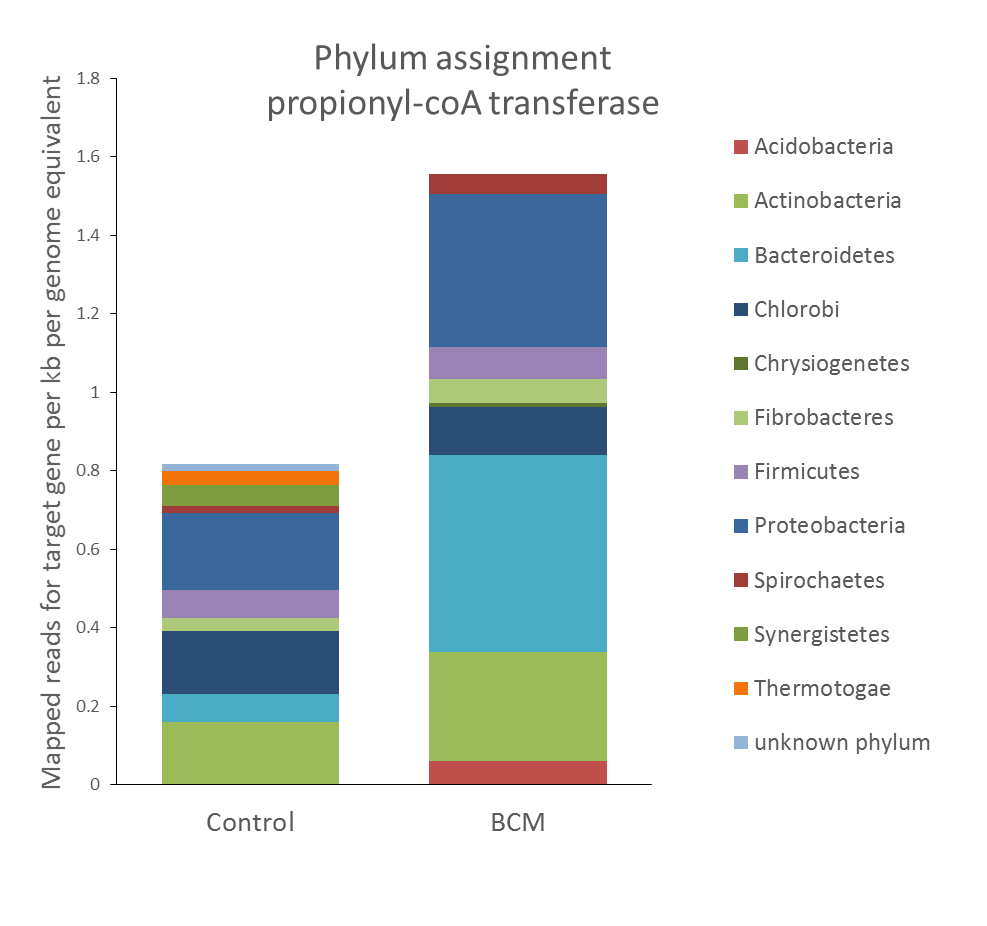
**Supplementary Figure 7**. Phylum level classification for reads assigend to e) propionyl-coA transferase pathway

**
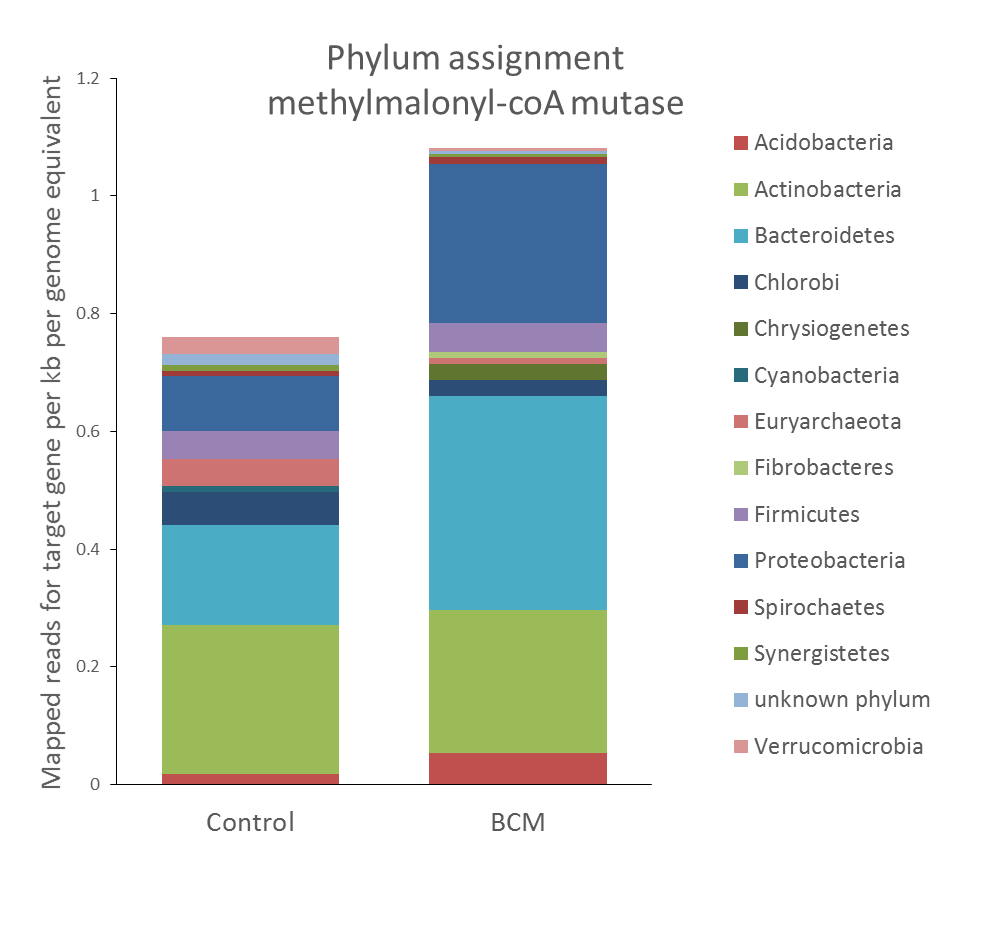
**

**Supplementary Figure 7**. Phylum level classification for reads assigend to f) methylmalonyl-coA mutase pathway


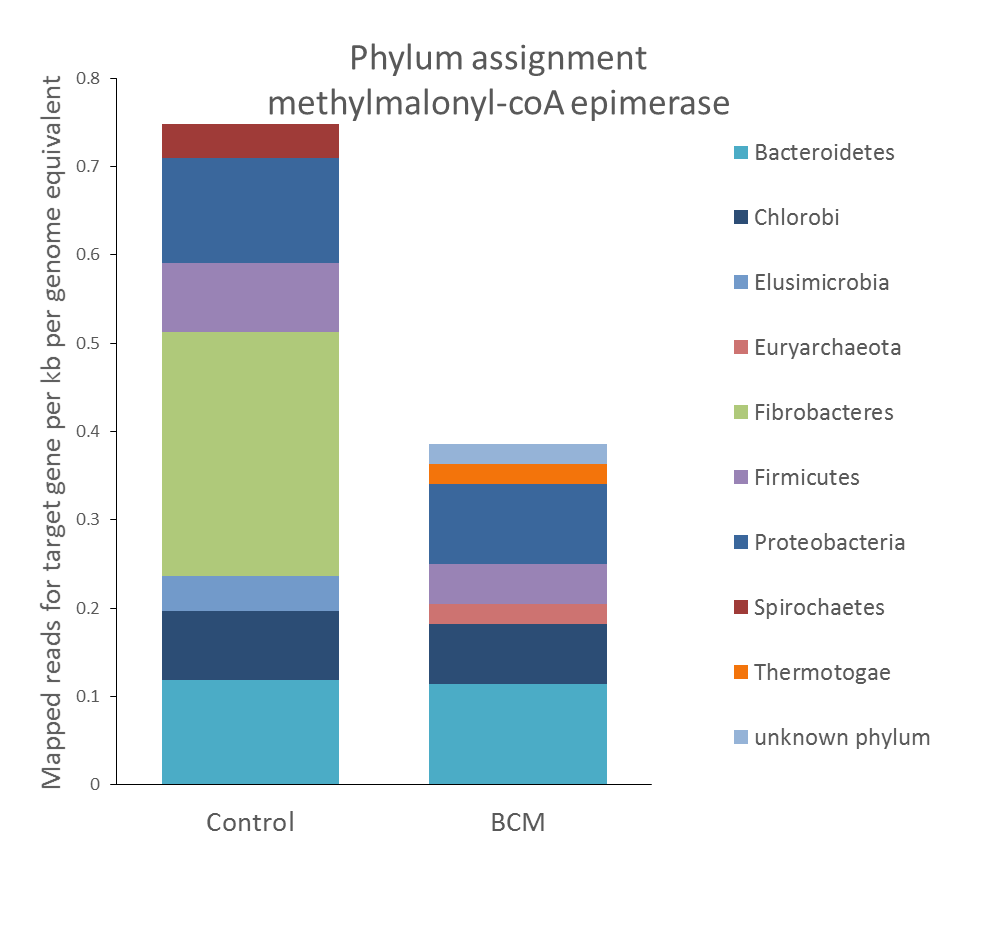


**Supplementary Figure 7**. Phylum level classification for reads assigend to g) methylmalonyl-coA epimerase pathway


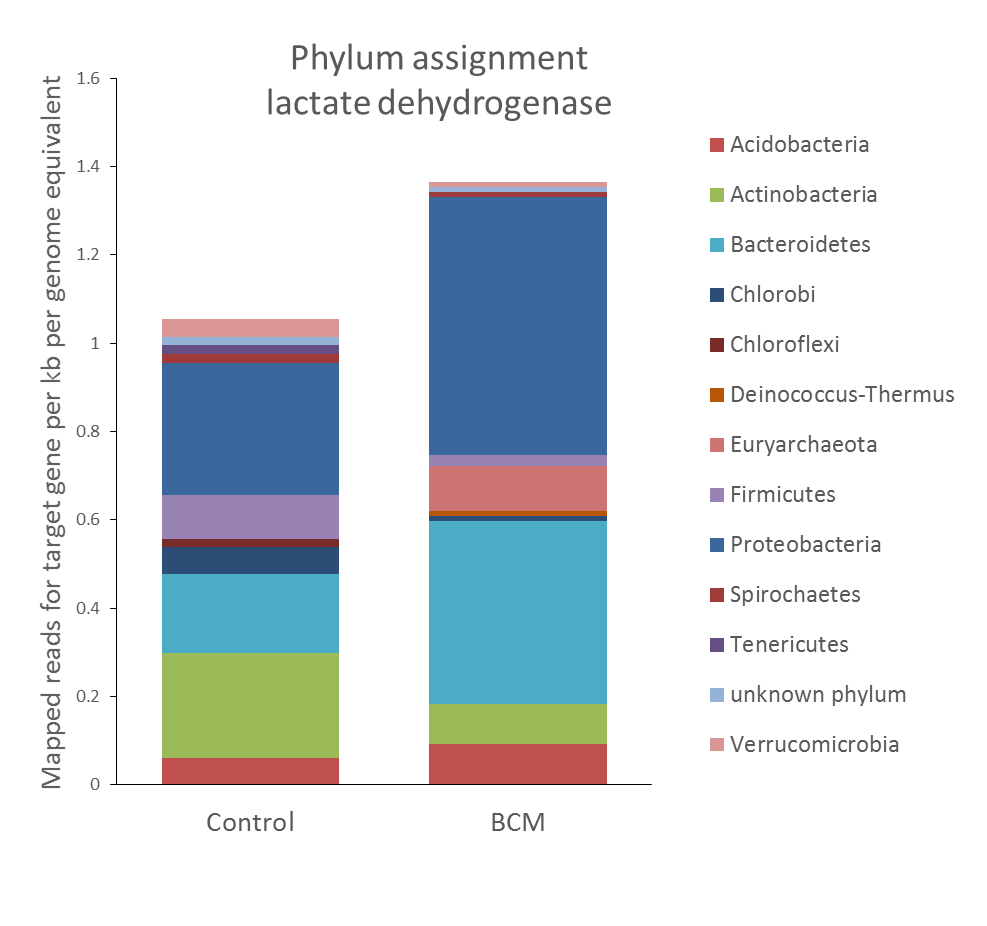


**Supplementary Figure 7**. Phylum level classification for reads assigend to h) lactate dehidrogenase pathway
